# An Antibody Persistent and Protective Two rSsCLP-Based Subunit Cocktail Vaccine against *Sarcoptes scabiei* in a Rabbit Model

**DOI:** 10.3390/vaccines8010129

**Published:** 2020-03-16

**Authors:** Nengxing Shen, Wenrui Wei, Yuhang Chen, Yongjun Ren, Lang Xiong, Yuanyuan Tao, Xiaobin Gu, Yue Xie, Xuerong Peng, Guangyou Yang

**Affiliations:** 1 Department of Parasitology, College of Veterinary Medicine, Sichuan Agricultural University, Wenjiang 611130, China; shennengxing@stu.sicau.edu.cn (N.S.); wenruiwei1995@stu.sicau.edu.cn (W.W.); s20177540@stu.sicau.edu.cn (Y.C.); xionglang@stu.sicau.edu.cn (L.X.); taoyuanyuan@stu.sicau.edu.cn (Y.T.); 13720@sicau.edu.cn (X.G.); 14265@sicau.edu.cn (Y.X.); 2 Animal Breeding and Genetics Key Laboratory of Sichuan Province, Chengdu 610066, Sichuan, China; s2018303036@stu.sicau.edu.cn; 3 Department of Chemistry, College of Life and Basic Science, Sichuan Agricultural University, Wenjiang 611130, China; 10533@sicau.edu.cn

**Keywords:** scabies, *Sarcoptes scabiei*, chitinase like-proteins, sub-unit cocktail vaccine, persistent antibody, immune-protection

## Abstract

Scabies is a highly contagious disease caused by *Sarcoptes scabiei* which burrows into stratum corneum of host’s skin. In this study, after optimizing vaccination schedule, a vaccination trial is comprised of three test groups of rabbits (*n* = 10/group) by immunization with (1) rSsCLP5; (2) rSsCLP12; or (3) a mixture of rSsCLP5 and rSsCLP12, three biological replicates groups (*n* = 10/group) and three control groups (*n* = 10/group). Levels of specific IgG, total IgE and cytokines in sera were detected and histopathologically analyzed as indicators of vaccine effects. The results showed that 85% (17/20) of rabbits exhibited no detectable skin lesions of *S. scabiei* infestation in mixed protein groups compared to single protein groups with 75% (15/20) and 70% (14/20), respectively. Moreover, the deworming rates of mixed groups are increased by 10%–20% compared with that of single groups. Each of six groups immunized with rSsCLP displayed significant increases of specific IgG, total IgE, IL-10, and TNF-α. The degree of skin damage in test groups also significantly lower than that of control groups. Thus, purified rSsCLP5 and rSsCLP12 subunit cocktail vaccine induced robust immune protection and could significantly decrease mite populations to reduce the direct transmission between rabbits.

## 1. Introduction

Scabies is a highly contactable contagious infection of the skin caused by the etiological agent, *Sarcoptes scabiei*, burrowing into the stratum corneum of the host’s skin [1,2]. Moreover, scabies was added to the World Health Organization (WHO) Neglected Tropical Diseases portfolio in 2017 [3]. Worldwide, more than 100 species of mammals suffer from sarcoptic mange infestation caused by *S. scabiei* [4]. It was also reported that approximately 100 million individuals suffer from an infestation of the scabies mite throughout the world [5]. In addition, as a global disease, scabies is widely distributed in indigenous populations [6] and impoverished regions of developing countries [5,7,8] (e.g., Pacific and regions of Latin America). Among vulnerable populations, the prevalence of scabies is substantially higher in children than in adolescents and adults [5,8]. Moreover, if left untreated, scabies can lead to a series of secondary infections and complications (e.g., serious pyoderma [9], rheumatic heart disease (RHD), and acute poststreptococcal glomerulonephritis (APSGN)) [10]. Therefore, the high prevalence and serious harm induced by scabies is a great threat to human health and increases the financial burden of public medical institutions [11].

Scabies is historically a challenging global infectious disease, for which acaricides are currently used as measures of controlling the scabies mite [12,13]; however, the unreasonable use of acaricides can lead to the development of drug resistance, toxicity, and associated side-effects [14,15]. Therefore, the development of a vaccine remains a high priority for global research. Unfortunately, there is currently no available effective commercial vaccine, and although some protein-based candidate studies have been reported [16,17], most of which have resulted in no protective effects; thus, an alternative anti-mite vaccine is required. 

After an infestation of *S. scabiei*, hosts have been shown to exhibit protective immune responses since an *S. scabiei* infestation can induce both the innate and adaptive immune responses in the host [18]. Such responses have a meaningful impact on developing a vaccine to control this mite. With the development of biological product technology, and the completion of the omics data [19,20,21], various types of recombinant subunit vaccines have been generated. Currently, few mite’s vaccine studies have been published. One study found no protective effect when goats were immunized with a scabies mite extract [22]. Moreover, rabbits immunized with the recombinant mites’ antigens, Ssag1 and Ssag2, produced high levels of antibodies; however, the rabbits were not protected from *S. scabiei* challenge or decreased mite burden [23]. However, in our previous study conducted in 2018 [24], we found that 74.3% (26/35) of rabbits immunized with rSsCLP5 displayed no detectable lesions following a challenge with *S. scabiei*. In addition, the mite burden in the test groups was significantly lower than that in the control groups.

Chitinase-like proteins (CLPs) and chitinases are part of a family of proteins that are increasingly associated with infection, T cell-mediated inflammation, wound healing, allergy, and asthma [25]. In preliminary studies rSsCLP5 and rSsCLP12, recombinant proteins representing two CLPs from *S. scabiei*, were found to exhibit good immunogenicity [24,26]. In the present study, based on single candidate protein vaccine studies, the vaccination schedule was optimized to obtain the best procedure and compared the effects of single protein vaccines with the use of a combined candidate protein vaccine. These findings will demonstrate the advantages of a subunit cocktail vaccine in improving immune protection, and provides further insight into achieving higher immune protection in anti-mite vaccine research.

## 2. Materials and Methods

### 2.1. Ethics Approval and Consent to Participate

The animal study was reviewed and approved by the Animal Care and Use Committee of Sichuan Agricultural University (SYXK 2014-187). All animal procedures used in this study were carried out in accordance with Guide for the Care and Use of Laboratory Animals (National Research Council, Bethesda, MD, USA) and recommendations of the ARRIVE guidelines [27]. All methods were carried out in accordance with relevant guidelines and regulations.

### 2.2. Source of Mites and Animals

A total of 210, three-month-old scabies-free New Zealand white rabbits (*Oryctolagus cuniculus*) weighing 2.3 kg ± 0.2 kg were purchased from Chengdu Tatsuo Biological Technology Co., Ltd. (Chengdu, China), of which 108 (54 females and 54 males) rabbits were used to complete the optimized vaccination schedule. Twelve rabbits (six females and six males) were infested using direct contact for one month with seeder rabbits to simulate the natural process of infestation. After one month, live mites (adults, nymphs, and larvae) were collected from the infested rabbits to perform a challenge test. The remaining 90 rabbits (45 females and 45 males) used for the vaccination trial were housed individually in 0.3 m^2^ wire cages, with strict acaricide processing, in rooms with no associated mite-animal breeding history. All of the rabbits were provided with food and water ad libitum, and each of the housed rooms had good air conditioning and ventilation.

### 2.3. Preparation of Proteins for Vaccination

The recombinant protein contains the inserted target gene fragments (CLP5 or CLP12), Trx-tag, His-tag and S-tag. The protein expressed by pET-32a (+) vector without inserting target gene fragment is called Trx-His-S tag protein. For the expression of recombinant proteins, the soluble rSsCLP5 protein, the inclusion rSsCLP12 protein and the pET-32a (+) Trx-His-S tag protein were prepared as before described [24,26]. Soluble rSsCLP5 protein and Trx-His-S tag protein were purified under non-denaturing conditions. Briefly, after the expression completed, *Escherichia coli* cells were collected by centrifugation at 7000 rpm at 4 °C for 10 min, resuspended in 50 mM Tris-HCL (pH 7.6) and sonicated in ice water mixture. All the following steps of purification were performed at 4 °C. After sonication of cells and centrifugation (12,000 rpm at 4 °C for 15 min). Protein rSsCLP5 and Trx-His-S tag protein were further purified by a Ni-NTA His-tag affinity chromatography kit (Bio-Rad Laboratories, Hercules, CA, USA): soluble protein was diluted 1:1 with binding buffer (50 mM NaH_2_PO_4_, 300 mM NaCl, 5 mM imidazole, pH 8.0) and bound to a pre-equilibrated Ni-NTA His-tag affinity chromatography kit (Bio-Rad Laboratories, Hercules, CA, USA). The kit was washed with 5 column volumes of binding buffer (50 mM NaH_2_PO_4_, 300 mM NaCl, 5 mM imidazole, pH 8.0) to wash away unbound proteins. Bound proteins were eluted using elution buffer (50 mM NaH_2_PO_4_, 300 mM NaCl, 150 mM imidazole, pH 8.0) and collected in tubes. Then, the column was eluted with high-concentration elution buffer (50 mM NaH_2_PO_4_, 300 mM NaCl, 400 mM imidazole, pH 8.0) in order to clean it. After equilibrating it with binding buffer (50 mM NaH_2_PO_4_, 300 mM NaCl, 5 mM imidazole, pH 8.0), it was finally eluted with 20% alcohol and the column was stored with 20% alcohol. Purification of inclusion rSsCLP12 protein should be purified under denaturing conditions with 8M urea. That is, all purification-related solutions require the addition of 8 M urea. The other steps are the same as purification method described above. After purification, the salts were removed by dialysis and the proteins were analyzed by SDS-PAGE. All proteins were stored at −80 °C until further use.

### 2.4. Optimization of a Vaccination Schedule

To screen for the optimal schedule with high antibody titers and long-lasting antibody levels, the optimization of the vaccination schedule was conducted as described in Table S1 (see Supplementary Materials). Serum samples were collected prior to vaccination and each month during the experiment. An rSsCLP5-based ELISA [24] and rSsCLP12-based ELISA [26] were used to detect the peak antibody titer and duration. All of the sera samples were stored at −20 °C until further use.

### 2.5. Vaccination Trial and Mite Challenge

According the optimized vaccination schedule, in the vaccination trial, a total of 90 rabbits (45 females and 45 males) were randomly divided into nine groups, with five females and five male rabbits in each group. All of the rabbits were immunized twice at 15-day intervals (the best schedule) by a subcutaneous injection in the neck. The first group was injected with 1 mL 0.01 M PBS (137 mM NaCl, 2.7 mM KCl, 10 mM Na_2_HPO_4_, 2 mM KH_2_PO_4_, pH 7.4) as unvaccinated controls; the second group was inoculated with 1 mL Quil-A saponin adjuvant (Sigma, USA) at a concentration of 1 mg/mL (dissolved in PBS) as an adjuvant control; the third group was immunized with 100 μg (initial injection and second injection) purified protein from the pET-32a (+) Trx-His-S tag protein with 1 ml Quil-A saponin at a concentration of 1 mg/mL (dissolved in PBS) as Trx-His-S tag protein controls; the fourth group was immunized with 100 μg purified rSsCLP5 protein with 1 mL Quil-A saponin at a concentration of 1 mg/mL (dissolved in PBS) for the first and second immunizations, respectively; the fifth group received the same immunization as the fourth group and served as biological replicates; the sixth group was immunized using the same methods as group four but was injected with the rSsCLP12; the seventh group received the same immunization as the sixth group and served as biological replicates; the eighth group was immunized twice at a 15-day interval with 100 μg rSsCLP5 protein and 100 μg rSsCLP12 with 1 mL Quil-A saponin at a concentration of 1 mg/mL (dissolved in PBS); and the ninth group received the same immunization as the eighth group and served as biological replicates.

**Figure 1:**
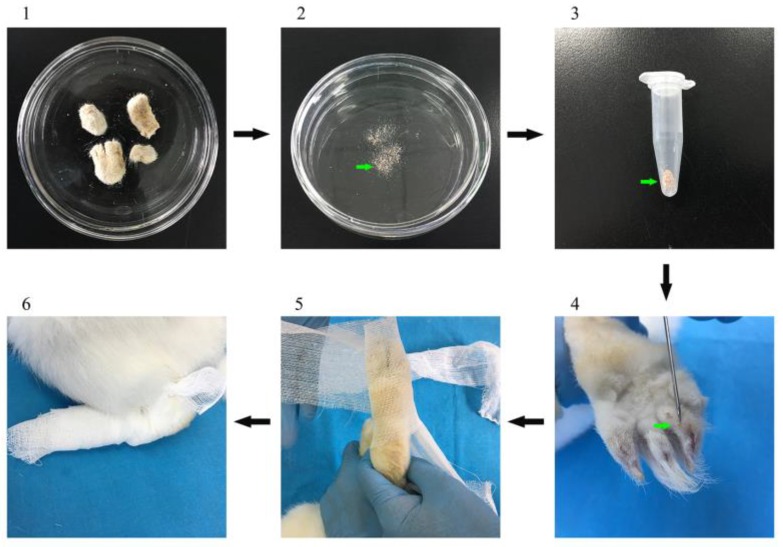
The collection of *S. scabiei* and mite challenge. (**1**) The skin scrapings from euthanized rabbits were collected and incubated at 38 °C; (**2**) the mites were counted after a 2-h incubation; (**3**) the mites were collected into a pipe; (**4**–**6**): rabbits were challenged via the two hind limbs.

Two weeks after the second vaccination, 12 previously infested rabbits were euthanized to collect the mites from the scraping skin (Figure 1). After the mites were collected and counted, the challenge was carried out immediately. Each rabbit was challenged with approximately 2000 live mites onto the two hind limbs. The challenge area was selected because mange lesions in naturally infested rabbits are most frequently initially observed in the hind feet (Figure 1). Serum samples were collected prior to vaccination and every week during the experiment until 4 weeks post-infection (wpi). All serum samples were stored at −20 °C until further use.

### 2.6. Clinical Monitoring and Mite Burden

During the vaccination trial, mite-free animals before infestation and skin lesions of the hind legs of infected rabbits were photographed after challenge. Mange lesion areas and the body weights were measured during the vaccination trial on a weekly basis. After the challenge, skin lesions caused by the mites were assessed at weekly intervals from 1 wpi to 4 wpi. The lesion areas measured using a caliper and the inflammatory reaction and skin lesions were graded as follows: 0—no inflammatory reaction; 1—mild inflammatory reaction; 2—severe inflammatory reaction; 3—lesions first observed on the limbs (lesions ≤ 7.75 cm^2^); 4—lesions between 7.75 cm^2^—15.5 cm^2^ (including 15.5 cm^2^) and 5—lesions between 15.5 cm^2^—31 cm^2^ [16,28]. Four weeks after challenge, the rabbits were euthanized and the hind limbs with crusts were collected to count the number of mites [24,29].

### 2.7. Measurement of Antibody and Cytokine Responses

The serum samples collected from the vaccination trial were analyzed for the presence of specific IgG antibodies with a rSsCLP5-based indirect ELISA [24] and rSsCLP12-based indirect ELISA [26], total IgE antibodies with an ELISA kit (Elabscience, Wuhan, China), and the level of IL-4, IL-10, IFN-γ, and TNF-α with an ELISA kit (Elabscience, Wuhan, China). The indirect ELISA procedures were performed as previously described and the total IgE and cytokine ELISA procedures were performed in accordance with the manufacturer’s instructions.

### 2.8. Histology of Skin Lesions

The skins lesion on the hind limbs obtained from euthanized rabbits were collected to detail the skin integrity, extent of injury, thickness of the hyperplasia, and aggregation of inflammatory cells. For histological analysis, the samples were fixed in 4% paraformaldehyde, embedded in paraffin, sectioned at a thickness of 5 μm, and stained with hematoxylin and eosin (H&E) and toluidine blue.

### 2.9. Statistical Analyses

All statistical tests were performed with SPSS 16.0 and all data were analyzed by treating the immunized group and time as fixed factors. The rabbits were considered as random factors to account for repeated measures variability. The one-sample Kolmogorov-Smirnov test was performed to determine whether the paired-difference variables of OD 450 nm for IgG, IgE, and cytokine values were normally distributed at same time point within each group. The mean ± SD of the data were presented in the appropriate sections. The Duncan Multiple range test with the alpha value set as 0.05, was applied to compare the level of IgG, IgE, and cytokines, as well as the skin lesion scores at different time points within the same infestation group, and at the same time point between the different infestation groups. 

## 3. Results

### 3.1. Vaccination Trial and Clinical Monitoring

According to the optimal vaccine formulations and approaches described in the supplementary results (see Supplementary Materials), the optimal number of immunizations was two, at an interval of 15 days, with an optimal single recombinant protein immunization dose of 100 μg/mL (100 μg purified rSsCLP5/rSsCLP12 protein with 1 mL Quil-A saponin) at each administration. The optimal mixed protein immunization dose was 200 μg/mL (100 μg purified rSsCLP5 protein and 100 μg purified rSsCLP12 protein with 1 mL Quil-A saponin) at each administration (Bold sections of Table S1).

**Figure 2:**
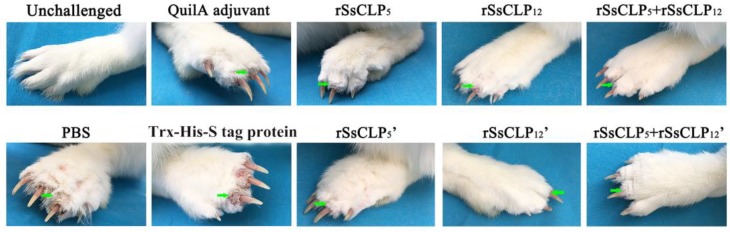
Clinical symptoms in rabbits after mite challenge for four weeks. unchallenged, the rabbits free of mite infestation; PBS, vaccinated with 1 mL 0.01 M PBS each time; QuilA adjuvant, 1 mL QuilA saponin at a concentration of 1 mg/mL (dissolved in PBS) each time; Trx-His-S tag protein, vaccinated with 100 μg (first time) and 100 μg (second time) purified pET-32a (+) Trx-His-S tag protein with 1 mL QuilA saponin at a concentration of 1 mg/mL (dissolved in PBS); rSsCLP_5_, rSsCLP_5_’, rSsCLP_12_, and rSsCLP_12_ refer to the test groups immunized with 100 μg purified rSsCLP5 or rSsCLP12 protein with 1 ml Quil-A saponin at a concentration of 1 mg/mL (dissolved in PBS) for the first and second immunizations, respectively; rSsCLP_5_ + rSsCLP_12_ and rSsCLP_5_ + rSsCLP_12_’ refer to the test groups immunized with the mixture of 100 μg rSsCLP5 protein and 100 μg rSsCLP12 with 1 mL Quil-A saponin at a concentration of 1 mg/mL (dissolved in PBS) for two immunizations. The rSsCLP_5_ and rSsCLP_12_ refer to rSsCLP5 and rSsCLP12, respectively.

After the challenge, the grades of the inflammatory reaction were recorded each week, and there were extremely significant differences between the six test groups and three control groups from 2 wpi until the end of the experiment at 4 wpi (*p* < 0.01; Table 1). The itching and inflammatory symptoms appeared at five days post-infestation (dpi) in the experimental rabbit population. Interesting, each of the nine groups displayed inflammatory reactions at 1 wpi. At 10 dpi, the clinical symptoms in two single vaccinated groups manifested in approximately 70% of the rabbits weakened or disappeared and showed no significant differences compared to the mite free rabbits. However, only three rabbits exhibited obvious clinical symptoms in the two mixed groups, and the remaining 85% of rabbits were symptom-free at 10 dpi. Also, the number of symptomatic rabbits in mixed protein groups were lower than that in single protein groups from two to four weeks after challenge (Table 1). As the infestation progressed, the symptoms in the three control groups became increasingly more serious. Most of the rabbits in the four single protein groups and two mixed protein groups were in a healthy state free from mange, and a minority of the rabbits exhibited mild mange that was significantly lower than the controls from 1 wpi until the end of the experiment at 4 wpi (Figure 2). At the end of the experiment, all rabbits showed an increase in body weight from 0.3 kg to 0.55 kg at 4 wpi (data not shown), and the disease did not spread to the forelimbs.

### 3.2. Lesion Size and Mite Burden

**Figure 3:**
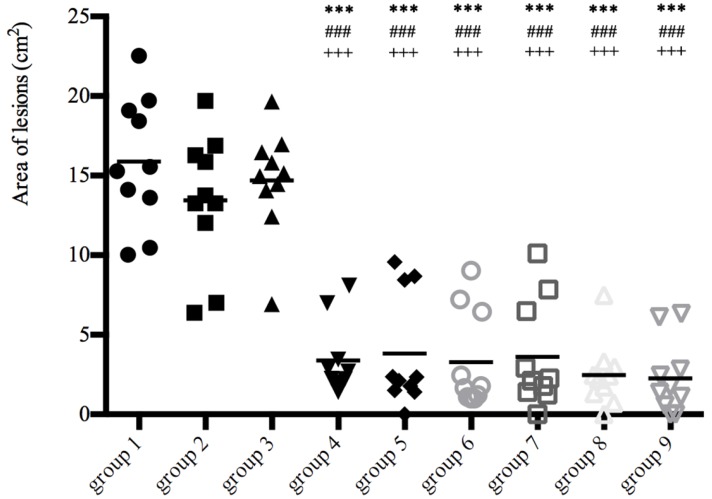
Lesion areas of the rabbit hind limbs at four weeks post-mite challenge. Group 1, vaccinated with 1 mL 0.01 M PBS each time; group 2, vaccinated with 1 mL QuilA saponin at a concentration of 1 mg/mL (dissolved in PBS) each time; group 3, vaccinated with 100 μg (first time) and 100 μg (second time) purified pET-32a (+)Trx-His-S tag protein with 1 mL QuilA saponin at a concentration of 1 mg/mL (dissolved in PBS); group 4, group 5, group 6, and group 7 refer to the test groups immunized with 100 μg purified rSsCLP5 or rSsCLP12 protein with 1 mL Quil-A saponin at a concentration of 1 mg/mL (dissolved in PBS) for the first and second immunizations, respectively; group 8 and group 9 refer to the test groups immunized with the mixture of 100 μg rSsCLP5 protein and 100 μg rSsCLP12 with 1 mL Quil-A saponin at a concentration of 1 mg/mL (dissolved in PBS) for the two immunizations. The lesion areas in the rSsCLP immunized groups (rSsCLP5, rSsCLP12, and rSsCLP5 + rSsCLP12) were significantly lower than that in the PBS control (*** *p* < 0.01), Quil A control (### *p* < 0.01) and Trx-His-S tag protein control (+++ *p* < 0.01).

The protective effects of the rSsCLP-based vaccine were also assessed by measuring the lesion area after challenge (Figure 3). The grades of the lesion area show that there were extremely significant differences between the six test groups and three control groups after 4 wpi (*p* < 0.01; Figure 3), and the differences became more apparent over the course of the infestation. The mite burden was detected as an important indicator to evaluate the effects of the candidate proteins. Large differences in the number of mites revealed more than 4000 mites in each control group, but near 1500 mites in the rSsCLP5 and rSsCLP12 test groups, respectively (*p* < 0.05; Table 2). In particular, the mean detectable mite number was 781 and 637 of mixed protein groups have significant differences compared with three control groups (*p* < 0.05; Table 2), but no significant difference compared with single protein groups (*p* > 0.05; Table 2). Two mixed protein groups have 83.45% and 86.51% of the mite reduction rate respectively, but both the rates of the two single protein groups was nearly 70% (Table 2). In particular, the reduction rates of mixed protein groups are increased by 10%–20% compared with the reduction rates of single groups.

### 3.3. Antibody and Cytokine Responses

**Figure 4:**
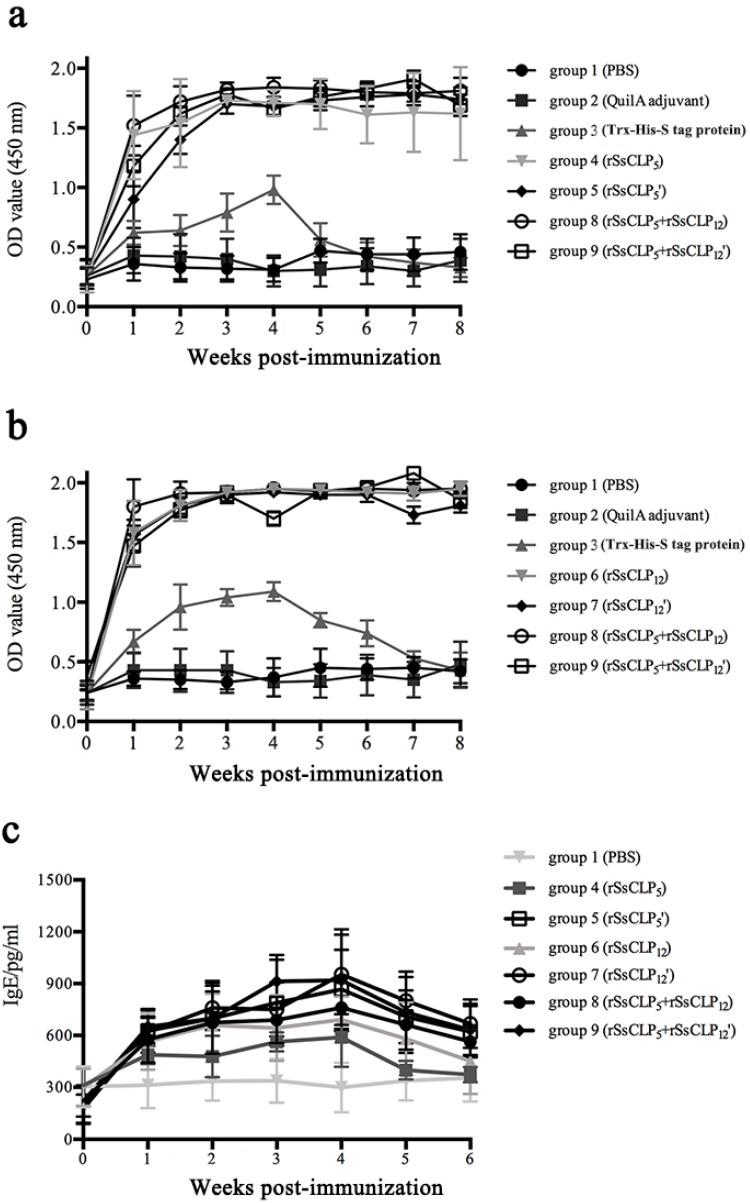
Variation of specific IgG (**a** and **b**) and total IgE (**c**) levels in the sera of immunized rabbits. a: the OD 450 nm value of the specific IgG antibodies was detected by an rSsCLP5-based indirect ELISA; b: the OD 450 nm value of specific IgG antibodies was detected by an rSsCLP12-based indirect ELISA; c: the total IgE antibody concentration (pg/mL) was detected by an ELISA kit (Elabscience, Wuhan, China). The rSsCLP_5_ and rSsCLP_12_ refer to rSsCLP5 and rSsCLP12, respectively.

The results of the specific IgG and total IgE antibody responses are shown in Figure 4. Prior to immunization, the values were low (OD450 nm < 0.15 for IgG and < 300 pg/mL for IgE) in all rabbits. After the primary vaccination, most of the rabbits in the test groups developed specific serum IgG and total IgE antibodies and the levels were extremely significantly higher than the antibody levels in the QuilA saponin and PBS control groups (*p* < 0.01; Figure 4). A significant increase in the IgG levels was first detected one week post-immunization, then subsequently increased strongly and peaked at two weeks after the final vaccination, after which the OD values plateaued. A significant increase in the total IgE level was also first detected one week post-immunization and progressively increased. The level of total IgE showed a rapid and strong increase during the first three weeks and peaked two weeks after the last immunization. The level of IgG and IgE antibodies was also observed in the Trx-His-S tag protein group, but were lower than that of the rSsCLP vaccine groups (*p* < 0.01; Figure 4). In the QuilA saponin and PBS control groups, the ELISA OD values of the specific IgG remained low throughout the experiment (Figure 4). The low antibody levels in the Trx-His-S tag protein group rapidly decreased during the experimental period (Figure 4).

**Figure 5:**
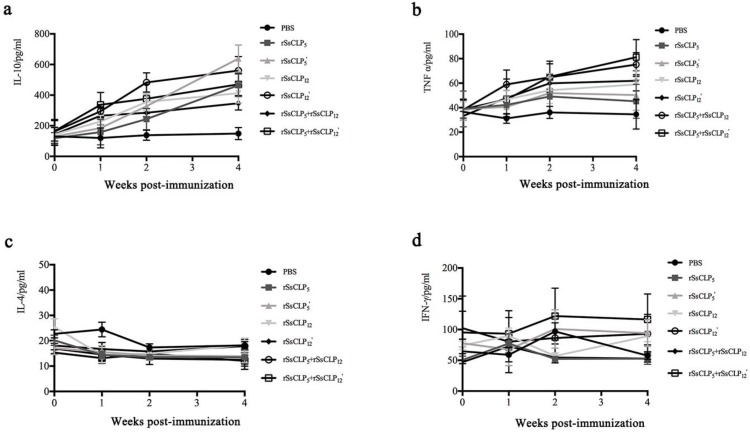
Variation of cytokines IL-10 (**a**), TNF-α (**b**), IL-4 (**c**), and IFN-γ (**d**) in the sera of immunized rabbits. a, the concentration of IL-10 (pg/mL) was detected by an ELISA kit (Elabscience, Wuhan, China); b, the concentration of TNF-α (pg/mL) was detected with an ELISA kit (Elabscience, Wuhan, China); c, the concentration of IL-4 (pg/mL) was detected using an ELISA kit (Elabscience, Wuhan, China); d, the concentration of IFN-γ (pg/mL) was detected using an ELISA kit (Elabscience, Wuhan, China). The rSsCLP_5_ and rSsCLP_12_ refer to rSsCLP5 and rSsCLP12, respectively.

Cellular immune responses in the test and control groups were measured to explore the T helper 1 (Th1) (IL-10, IFN-γ, and TNF-α) and/or Th2 (IL-4)-mediated responses after immunization with the rSsCLP proteins. The level of IL-10 in the rabbit serum was significantly increased compared to the PBS controls following immunization (*p* < 0.01; Figure 5a). In addition, while an increase in the TNF-α levels was observed in the test groups, these rates were lower than the level of IL-10 (*p* < 0.01; Figure 5b). However, the level of IL-4 and IFN-γ did not increase after the first and second immunizations (Figure 5c,d).

### 3.4. Histopathology

**Figure 6:**
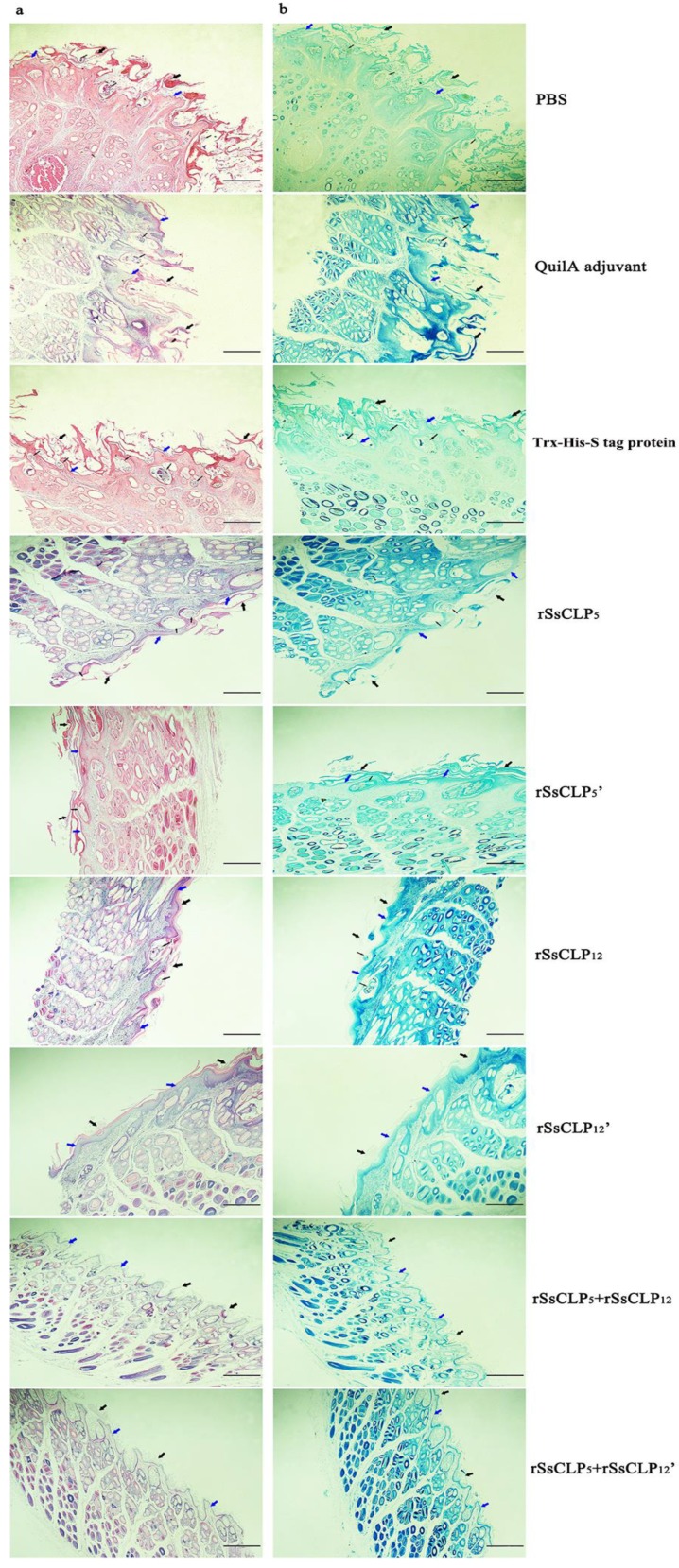
Histopathology of the hind limbs of the challenged rabbits. (**a**) stained with hematoxylin and eosin (H&E). (**b**) stained with toluidine. Thick blue arrows refer to the epidermis; thick black arrows refer to crusts caused by *S. scabiei*; thin black arrows refer to mites in the skin. The rSsCLP_5_ and rSsCLP_12_ refer to rSsCLP5 and rSsCLP12, respectively.

To examine the role of the rSsCLP5 and rSsCLP12-based vaccine in the development of disease, the lesion skins of the hind limbs was stained with hematoxylin and eosin (H&E) and toluidine, and examined using an optical microscope. The first three pictures in Figure 6a show the representative results of the histological analyses of the scabies skin lesions from the non-vaccinated rabbits. At 4 wpi, hyperplasia and thickening of the epidermis were evident and cellular infiltration was more marked in the control rabbits than in the vaccinated rabbits. In addition, the mixed proteins group significantly decreased the extent of skin injury and the aggregation of eosinophils (Figure 6a). There was no significant difference in the level of mast cell infiltration between each group (Figure 6b).

## 4. Discussion

The infestation of scabies mites will induce a variable immune response in the host [18,28], which provides a new perspective for anti-mite vaccine research. Recently, the total extract protein and recombinant proteins of mites were used as antigens to immunize animals and examine the effects of the vaccine [16,17]; however, no vaccines against scabies have been developed and research in this area is extremely limited [30]. Due to the global presence and severe disease caused by scabies infection, it is imperative to develop a global strategy for diagnosis and control [31].

Obvious clinical symptoms can be observed after rabbits infested by *S. scabiei* for 4 weeks [24,32]. However, it is different from scabies in human [33] or pigs [34] which takes longer to produce obvious symptoms. In our previous study, rSsCLP5-immunized rabbits induced high IgG antibody levels and 74.3% of the rabbits showed no detectable lesions following challenge with *S. scabiei* [24]. In addition, rSsCLP12 protein exhibits strong immunogenicity with a long duration of IgG antibodies in vaccinated rabbits. The vaccination program is closely related to the antibody level and the protective efficacy following immunization with the vaccine.

It is important to screen for an optimal vaccination schedule characterized by high antibody titers and long-lasting antibody levels. In scabies vaccine-related experiments, we first optimized the immune response to the candidate proteins, including the amount of immune protein, the number of immunizations, and the time interval for each immunization. In the present study, we did not conduct a challenge experiment since the main purpose was to screen for immune response that could obtain higher antibody levels and that persisted over longer periods. In some groups, the specific IgG antibody levels begin to decrease one month after the last immunization, but the levels in other groups could persist for three months or longer (Figure S1). In general, this significant difference indicates that the immunization program is an important factor for the protective efficacy of the vaccine. Immunization twice at 15-day intervals produce protective antibodies to scabies mites in rabbit, which is consistent with the immunization program that prime and boost immunization induced antisera to ticks in mice [35]. Moreover, two weeks after the first immunization, animals immunized with the same antigen develop a more robust and persistent antibody response [36]. Furthermore, immunization with 200 μg protein in the mixed proteins group is 100 μg rSsCLP5 and 100 μg rSsCLP12, which resulted in high immune efficacy that may be related to the intrinsic characteristics of the proteins and the physiological characteristics of the animals.

In this study, a mixed recombinant proteins vaccine was used to increase the antibody levels in the immunized animals, which may enhance the level of immune protection during mite challenge. In one study, sheep were immunized with a seven recombinant protein subunit cocktail vaccine against ovine psoroptic mange, which resulted in a highly significant reduction in both lesion size (up to 63%) and mite burden (up to 56%) at 6 wpi [37]. There was a substantial effect following the use of mixed proteins that may be related to the interaction between the proteins, antigen presentation, and superposition of immune effects. In our study, the number of symptomatic rabbits in the mixed protein groups were lower than that in the single protein groups from two to four weeks after challenge, especially the number of rabbits with crusts, and the lesion area of the mixed protein groups were significantly lower than that of the single protein groups (0.01 < *p* < 0.05), indicating that the use of mixed proteins can increase the resistance of rabbits to scabies mite infestation and has a superposition of immune effects. Moreover, the deworming rates of mixed groups are increased by 10%–20% compared with the deworming rates of single groups, which is a very big change in the research of scabies mite vaccines. In previous studies, following the immunization of rabbits [16,17] and sheep [37] with mite recombinant proteins or total crude protein, despite the induction of higher levels of antibodies, the experimental animals did not display a significant difference from the control group after mite challenge, suggesting that there was no protective immune response. With the use of mixed proteins in this study, high levels of specific IgG and total IgE antibody levels were induced to improve the level of immune protection following immunization. Some studies have shown that an absence of immune protection with a high level of IgG antibodies [17,23] may be related to the finding that some protein-induced IgG antibodies were not immune protective; however, the specific IgG antibodies produced in this study were immune protective in the challenged rabbits.

IgE is a type of immunoglobulin related to host animal resistance [38,39], which directly participates in the resistance of animals to bacteria, viruses, and parasites, especially regarding the regulation of allergies [40,41]. The present results show that rabbits can be induced immune response for a short period of time after immunization, characterized by producing high levels of IgE, with an antibody response that can persist following challenge. The produced antibodies can cause the animal to be in an immune-stimulated state causing rabbits resist mite challenge, indicating that the IgE antibodies are involved in the resistance to scabies. Some studies have found no immune-protection following immunization that generated an IgE antibody response, suggesting that the immune-protective properties of animals may be related to the coordination of IgG and IgE-specific antibody responses, rather than a separate or minor effect of a single antibody subtype. In addition, other studies have suggested that some vaccines may be involved in cellular immunity in immunized animals.

After the infestation of the scabies mite, cytokines and chemokines will increase or decrease to varying degrees at different time points [42,43]. At the same time, in vitro experiments show that the natural protein and some recombinant proteins can promote the increase or decrease of cytokines and chemokines [44,45], indicating that some proteins are involved in the regulation of cytokines and chemokines during the infestation. In the present study, cytokines were also used as an indicator of immune-protection. After immunization, we measured IL-4, IL-10, IFN-γ, and TNF-α, respectively. The results showed that the level of IL-10 and TNF-α in the six experimental groups were significantly increased one week post-immunization, which continued until the rabbits were challenged. In addition, the cytokine levels in the mixed protein group were higher than that in the single protein groups (*p* < 0.01), indicating that rSsCLP5 and rSsCLP12 are closely related to the production of IL-10 and TNF-α, and that the two proteins may be involved in the immunomodulation of these cytokines. Moreover, both IL-10 and TNF-α play an important role in the Th1 cytokine response, indicating that the Th1 immune response is involved in the immune protection of rabbits against scabies.

*S. scabiei* burrow into the stratum corneum of the host skin and further directly destroy the skin, causing allergies and a range of immune responses. Histological observations show that the skin on the foot of the challenged rabbits was severely damaged and extreme hyperplasia was evident in the control groups. In addition, mites were present in the epidermis and severe eosinophil infiltration was observed around the sections. However, in the vaccinated groups, the degree of skin damage and hyperplasia was minor compared to the controls. Furthermore, no mites were detected in the epidermis and only moderate eosinophil infiltration was present. In general, immune response induced by mixed proteins could protect the integrity of rabbit’s skin and reduce inflammation.

## 5. Conclusions

In the present study, the optimization of the vaccination schedule is essential to have a meaningful impact on the development of an effective anti-mite vaccine. The purified rSsCLP5 and rSsCLP12 subunit cocktail vaccine induced strong protective immunity with the overlapping efficacy. Although vaccination with these two antigens did not provide complete resistance to mite infestation, the vaccines could significantly decrease the mite populations to reduce the level of direct transmission between rabbits. Thus, the combined use of candidate proteins may provide a significant advantage in the development of anti-mite vaccines.

## Figures and Tables

**Table 1:** Mean mange lesion scores at different time points in rabbits and the number of symptomatic and asymptomatic rabbits after challenged with *S. scabiei*.

Group	Prior to Challenge	Week 1 Post-Challenge	Week 2 Post-Challenge	Week 3 Post-Challenge	Week 4 Post-Challenge
group 1 (PBS) (*n* = 10)	0^a^ (0/10)	2.1 ± 0.88_a_^b^ (10/0)	1.7 ± 0.67_a_^b^ (10/0)	2.8 ± 1.14_a_^c^ (10/0)	4.5 ± 0.53_a_^d^ (10/0)
group 2 (QuilA adjuvant) (*n* = 10)	0^a^ (0/10)	1.7 ± 0.48_abc_^b^ (10/0)	1.9 ± 0.74_a_^b^ (10/0)	2.5 ± 0.71_a_^c^ (10/0)	4.2 ± 0.79_a_^d^ (10/0)
group 3 (Trx-His-S tag protein) (*n* = 10)	0^a^ (0/10)	1.6 ± 0.70_abc_^b^ (9/1)	1.7 ± 0.67_a_^b^ (10/0)	2.1 ± 0.57_a_^b^ (10/0)	4.3 ± 0.67_a_^c^ (10/0)
group 4 (rSsCLP_5_) (*n* = 10)	0^a^ (0/10)	2.0 ± 0.67_ab_^b^ (10/0)	1.2 ± 1.03_ab_^bc^ (3/7)	1.1 ± 0.74_b_^c^ (4/6)	1.7 ± 1.25_b_^bc^ (2/8)
group 5 (rSsCLP_5_’) (*n* = 10)	0^a^ (0/10)	1.4 ± 0.70_abc_^b^ (10/0)	1.1 ± 0.74_ab_^b^ (2/8)	1.2 ± 1.03_b_^b^ (3/7)	1.9 ± 1.66_b_^b^ (3/7)
group 6 (rSsCLP_12_) (*n* = 10)	0^a^ (0/10)	1.6 ± 0.70_abc_^b^ (10/0)	1.1 ± 1.10_ab_^b^ (4/6)	1.0 ± 0.82_b_^b^ (3/7)	1.6 ± 1.43_b_^b^ (3/7)
group 7 (rSsCLP_12_’) (*n* = 10)	0^a^ (0/10)	1.3 ± 0.82_bc_^b^ (10/0)	1.1 ± 0.88_ab_^b^ (2/8)	1.3 ± 1.16_b_^b^ (3/7)	1.4 ± 1.71_b_^b^ (3/7)
group 8 (rSsCLP_5_ + rSsCLP_12_) (*n* = 10)	0^a^ (0/10)	1.3 ± 0.67_bc_^b^ (10/0)	0.4 ± 0.70_b_^a^ (1/9)	0.7 ± 1.06_b_^ab^ (1/9)	1.2 ± 1.03_b_^b^ (1/9)
group 9 (rSsCLP_5_ + rSsCLP_12_’) (*n* = 10)	0^a^ (0/10)	1.1 ± 0.88_c_^b^ (10/0)	0.7±0.67_b_^ab^ (2/8)	1.1 ± 0.88_b_^b^ (2/8)	1.0 ± 1.05_b_^b^ (2/8)

**Note:** Values within a column for the same group followed by different superscript lowercase letters were significantly different compared with different weeks-post-infestation (wpi) (*p* < 0.05), while same superscript lowercase letters were not significantly different (*p* > 0.05). Values within a column for different groups followed by different subscript lowercase letters were significantly different compared with same wpi (*p* < 0.05), while same subscript lowercase letters were not significantly different (*p* > 0.05). The results of the mange lesion scores were expressed as the mean ± standard deviation (SD). Two numbers separated by slash in parentheses refer to the number of symptomatic and asymptomatic rabbits, respectively. The rSsCLP_5_ and rSsCLP_12_ refer to rSsCLP5 and rSsCLP12, respectively.

**Table 2:** Mean mange mite burden at four weeks post-challenge and mite reduction rate of the rabbits.

Group	Number of Mites	Mite Reduction Rate
group 1 (PBS) (*n* = 10)	4719 ± 1072.39a	--
group 2 (QuilA adjuvant) (*n* = 10)	4464 ± 1663.22a	5.41%
group 3 (Trx-His-S tag protein) (*n* = 10)	4139 ± 742.05a	12.30%
group 4 (rSsCLP_5_) (*n* = 10)	1423 ± 984.64b	69.85%
group 5 (rSsCLP_5_’) (*n* = 10)	1640 ± 1534.27b	65.24%
group 6 (rSsCLP_12_) (*n* = 10)	1280 ± 863.21b	72.87%
group 7 (rSsCLP_12_’) (*n* = 10)	1338 ± 985.67b	71.66%
group 8 (rSsCLP_5_ + rSsCLP_12_) (*n* = 10)	781 ± 298.54b	83.45%
group 9 (rSsCLP_5_ + rSsCLP_12_’) (*n* = 10)	637 ± 449.66b	86.51%

**Note:** Values within the column of number of mites for the different group followed by different lowercase letters were significantly different (*p* < 0.05), while the same lowercases letters were not significantly different (*p* > 0.05). The results of the number of mites are expressed as the mean ± standard deviation (SD). The rSsCLP_5_ and rSsCLP_12_ refer to rSsCLP5 and rSsCLP12, respectively.

## Supplementary Materials

The supplementary materials contain the results of the optimal vaccine formulations and approaches. The following are available online at https://www.mdpi.com/2076-393X/8/1/129/s1, Figure S1: the level specific IgG antibodies in the vaccination schedule optimization, Table S1: vaccination schedule optimization.
