# Supplementary material for: An Antibody Persistent and Protective Two rSsCLP-Based Subunit Cocktail Vaccine against Sarcoptes scabiei in a Rabbit Model
Source: Vaccines (Basel). 2020 Mar 16;8(1):129. doi: 10.3390/vaccines8010129 (PMC7157647; doi:10.3390/vaccines8010129)
Supplement: Supplementary file 1 [file vaccines-08-00129-s001.pdf]

Supplementary Materials

Results

The optimal vaccine formulations and approaches

In this immune response optimization experiment, the immunization time(s) was/were administered as closely as possible to determine the greatest level of antibody production with appropriate immunization program. Both a single immunization time and two immunization times were employed, with the two immunizations performed at 15-day and 30-day intervals. All three immunization methods revealed that the level of specific IgG antibodies peaked two weeks after the last immunization. The highest level of IgG antibodies in the groups immunized twice was higher than the highest level of IgG antibodies in the groups that received a single immunization. Moreover, the groups immunized twice maintained a high antibody level for at least three months. Although higher antibody levels were achieved the groups that were immunized twice, the antibody levels were not sustained to the same extent at the 30-day interval compared with the 15-day interval, and the antibody levels in each group later decreased (Figure S1). In a comprehensive comparison of the antibody levels in these groups, the program with twice immunizations was found to be optimal at an interval of 15 days, and the optimal single recombinant protein immunization dose was 100 μg/mL (100 μg purified rSsCLP5/rSsCLP12 protein with 1 mL Quil-A saponin) for each administration. The optimal mixed protein immunization dose was 200 μg/mL (100 μg purified rSsCLP5 protein and 100 μg purified rSsCLP12 protein with 1 mL Quil-A saponin) for each administration (Bold sections of Table S1).

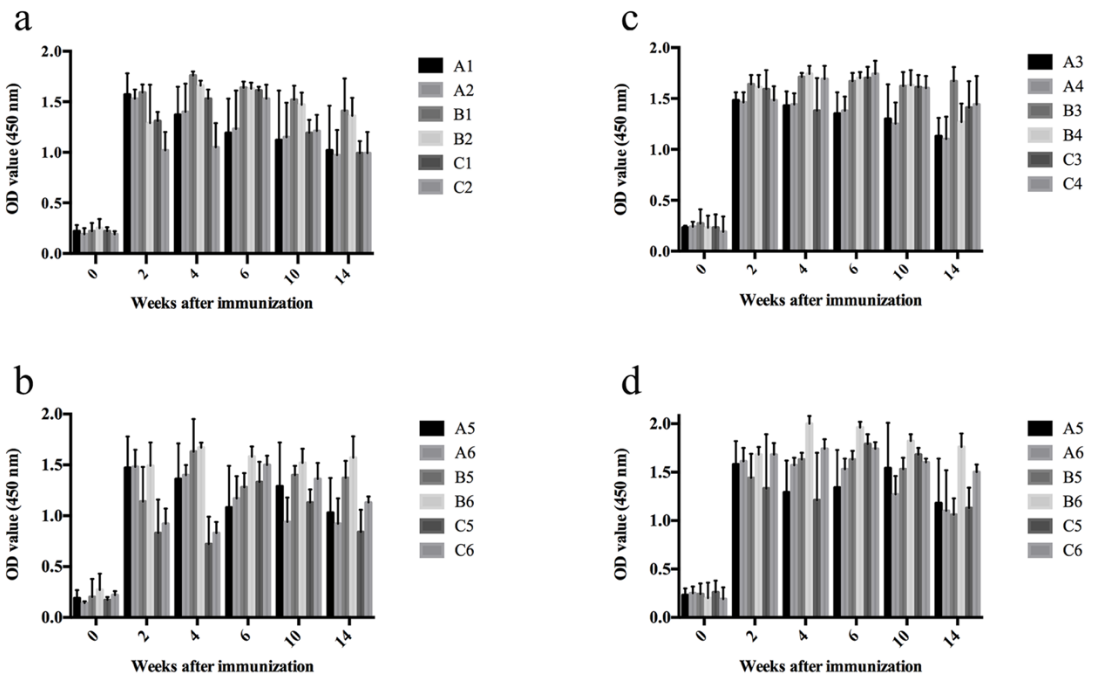

Figure S1. The level specific IgG antibodies in the vaccination schedule optimization. (**a**) and (**b**), the average OD 450 nm values detected by an rSsCLP5-based indirect ELISA; (**c**) and (**d**), the average OD 450 nm values detected by an rSsCLP12-based indirect ELISA. Group A1, A2, B1, B2, C1, and C2 immunized with single recombinant protein rSsCLP5. Group A3, A4, B3, B4, C3, and C4 immunized with single recombinant protein rSsCLP12. Group A5, A6, B5, B6, C5, and C6 immunized with mixed recombinant protein rSsCLP5 and rSsCLP12.

Table S1. Vaccination schedule optimization.

Groups	Animals	Inoculation Time(s)	Interval (Days)	Antigen Protein(s)	Protein Concentration (μg /mL)	
**A_1**	3♀+3♂	1	-	rSsCLP$_5$	200	-
A_2	3♀+3♂	1	-	rSsCLP$_5$	400	-
A_3	3♀+3♂	1	-	rSsCLP$_{12}$	200	-
A_4	3♀+3♂	1	-	rSsCLP$_{12}$	400	-
A_5	3♀+3♂	1	-	rSsCLP$_5$ + rSsCLP$_{12}$	100$_{(5)}$ + 100$_{(12)}$	-
A_6	3♀+3♂	1	-	rSsCLP$_5$ + rSsCLP$_{12}$	200$_{(5)}$ + 200$_{(12)}$	-
**B_1**	**3♀+3♂**	**2**	**15**	**rSsCLP$_5$**	**100**	**100**
B_2	3♀+3♂	2	15	rSsCLP$_5$	200	200
**B_3**	**3♀+3♂**	**2**	**15**	**rSsCLP$_{12}$**	**100**	**100**
B_4	3♀+3♂	2	15	rSsCLP$_{12}$	200	200
B_5	3♀+3♂	2	15	rSsCLP$_5$+ rSsCLP$_{12}$	50$_{(5)}$ + 50$_{(12)}$	50$_{(5)}$ + 50$_{(12)}$
**B_6**	**3♀+3♂**	**2**	**15**	**rSsCLP$_5$+ rSsCLP$_{12}$**	**100$_{(5)}$ + 100$_{(12)}$**	**100$_{(5)}$ + 100$_{(12)}$**
C_1	3♀+3♂	2	30	rSsCLP$_5$	100	100
C_2	3♀+3♂	2	30	rSsCLP$_5$	200	200
C_3	3♀+3♂	2	30	rSsCLP$_{12}$	100	100
C_4	3♀+3♂	2	30	rSsCLP$_{12}$	200	200
C_5	3♀+3♂	2	30	rSsCLP$_5$ + rSsCLP$_{12}$	50$_{(5)}$ + 50$_{(12)}$	50$_{(5)}$ + 50$_{(12)}$
C_6	3♀+3♂	2	30	rSsCLP$_5$ + rSsCLP$_{12}$	100$_{(5)}$ + 100$_{(12)}$	100$_{(5)}$ + 100$_{(12)}$

Note: 3♀+3♂ refer to three female rabbits and three male rabbits in each group. The rSsCLP$_5$ and rSsCLP$_{12}$ refer to rSsCLP5 and rSsCLP12, respectively. The subscripts 5 and 12 in parentheses refer to the rSsCLP$_5$ and rSsCLP$_{12}$, respectively. The group B_1, B_3, and B_6 in bold represent the optimal vaccine formulations and approaches.
